# Supplementary material for: Seasonal variation in microhabitat of salamanders: environmental variation or shift of habitat selection?
Source: PeerJ. 2015 Aug 13;3:e1122. doi: 10.7717/peerj.1122 (PMC4540018; doi:10.7717/peerj.1122)
Supplement: Supplemental Information 1 [file peerj-03-1122-s001.docx]

**Seasonal variation in microhabitat of salamanders: environmental variation or shift of habitat selection?**

Enrico Lunghi, Raoul Manenti, Gentile Francesco Ficetola

Supplemental Information

Supplemental Table S1: **Relationships between microhabitat features and a) presence of the species, b) presence of Adults and c) presence of Juveniles**. To take into account imperfect detection, this analysis assumes constant occupancy during winter (January-February) and summer (June-July) months; the species was considered present in a sector during one season if it was detected at least in one sampling occasion. As per-visit detection probability was 0.75 (see Results section), two visits allow to ascertain absence with 94% of confidence.

| Independent variable | *B* | χ^2^_1_ | *P* |
| --- | --- | --- | --- |
| a) All individuals |  |  |  |
|  |  |  |  |
| Season (winter/summer) |  | 4.61 | **0.032** |
| Humidity | -8.83 | 1.57 | 0.210 |
| Temperature | 0.25 | 0.01 | 0.905 |
| *Meta* | 0.79 | 0.06 | 0.801 |
| Light | -1.29 | 12.42 | **<0.001** |
| Season × humidity |  | 2.94 | 0.086 |
| Season × temperature |  | 12.47 | **<0.001** |
|  |  |  |  |
| b) Adults |  |  |  |
|  |  |  |  |
| Season (winter/summer) |  | 6.09 | **0.013** |
| Humidity | -4.93 | 0.05 | 0.823 |
| Temperature | 0.19 | 0.50 | 0.478 |
| *Meta* | -0.01 | 0.51 | 0.476 |
| Light | -1.95 | 7.90 | **0.005** |
| Season × humidity |  | 3.09 | 0.078 |
| Season × temperature |  | 11.08 | **<0.001** |
|  |  |  |  |
| c) Juveniles |  |  |  |
|  |  |  |  |
| Season (winter/summer) |  | 0.44 | 0.505 |
| Humidity | -35.55 | 3.72 | 0.053 |
| Temperature | 1.24 | 0.89 | 0.346 |
| *Meta* | 1.33 | 2.43 | 0.118 |
| Season × humidity |  | 1.24 | 0.266 |
| Season × temperature |  | 5.95 | **0.015** |

Supplemental Figure S1: **Preferences for temperature and humidity showed from analysis between contrasting periods: winter (Win) and summer (Sum).** Preferences are showed for (a-b) the species, for (c-d) Adults only and for (e-f) Juveniles only.

Supplemental Figure S2: **Violin plots representing temperature in cave sectors available (white) and occupied by cave salamanders (grey), during three months.**
